# Endodontic management of three-rooted maxillary second premolar in a patient with bilateral occurrence of three roots in maxillary second premolars

**DOI:** 10.4317/jced.50954

**Published:** 2012-12-01

**Authors:** Raju Chauhan, Shweta Singh

**Affiliations:** 1 BDS, MDS, Senior Lecturer. Department of Conservative Dentistry and Endodontics, Institute of Dental Sciences, Pilibhit Bypass Road, Bareilly, U.P., India; 2 BDS, MDS. Department of oral and maxillofacial pathology. Institute of Dental Sciences, Pilibhit Bypass Road, Bareilly, U.P., India

## Abstract

The possibility of bilateral maxillary second premolars having three separate roots is quite low; however, it must be taken into account in clinical and radiographic evaluations during endodontic treatment. A correct clinical and radiographic diagnosis based on knowledge of root canal anatomy and critical interpretation of radiographs is necessary for a safer and successful endodontic treatment of these teeth. Failure of root canal treatment can occur due to missed roots/canals and often diagnosed when patient experiences continuous post-operative pain and discomfort. This case report describes the root canal treatment of a three-rooted maxillary second premolar in a patient with bilateral occurrence of three roots in maxillary second premolars.

** Key words:**Anatomic variations, maxillary second premolar, radiculous.

## Introduction 

The success of endodontic treatment depends upon thorough debridement and complete obturation of the entire root canal system (1). The thorough knowledge of root canal space anatomy is a basic prerequisite for the successful completion of endodontic treatment (1), especially in cases where extra root canals are expected (2). Proper care and attention must be exercised in identifying and negotiating extra roots and canals (3). One of the challenging tasks facing clinicians is performing proper endodontic therapy for a maxillary second premolar because the root and canal system of these teeth can vary significantly among different racial and ethnic groups. Although three rooted maxillary premolars are rare but the possibility of extra roots or canals should be borne in mind to ensure successful endodontic treatment.

The literature reveals wide variations in root canal morphology of maxillary premolars. Pineda and Kuttler (4), could not find three rooted maxillary second premolar in their study. Vertucci (5) had reported an incidence of 1% of maxillary second premolars with three canals while Pecora et al (6) reported 0.3%. There are few case reports in the literature which reported the bilateral occurrence of three rooted maxillary second premolars. This case reports the endodontic management of a three rooted maxillary second premolar in a patient with bilateral occurrence of three separate roots in maxillary second premolars.

## Case Report

**Figure 1:**
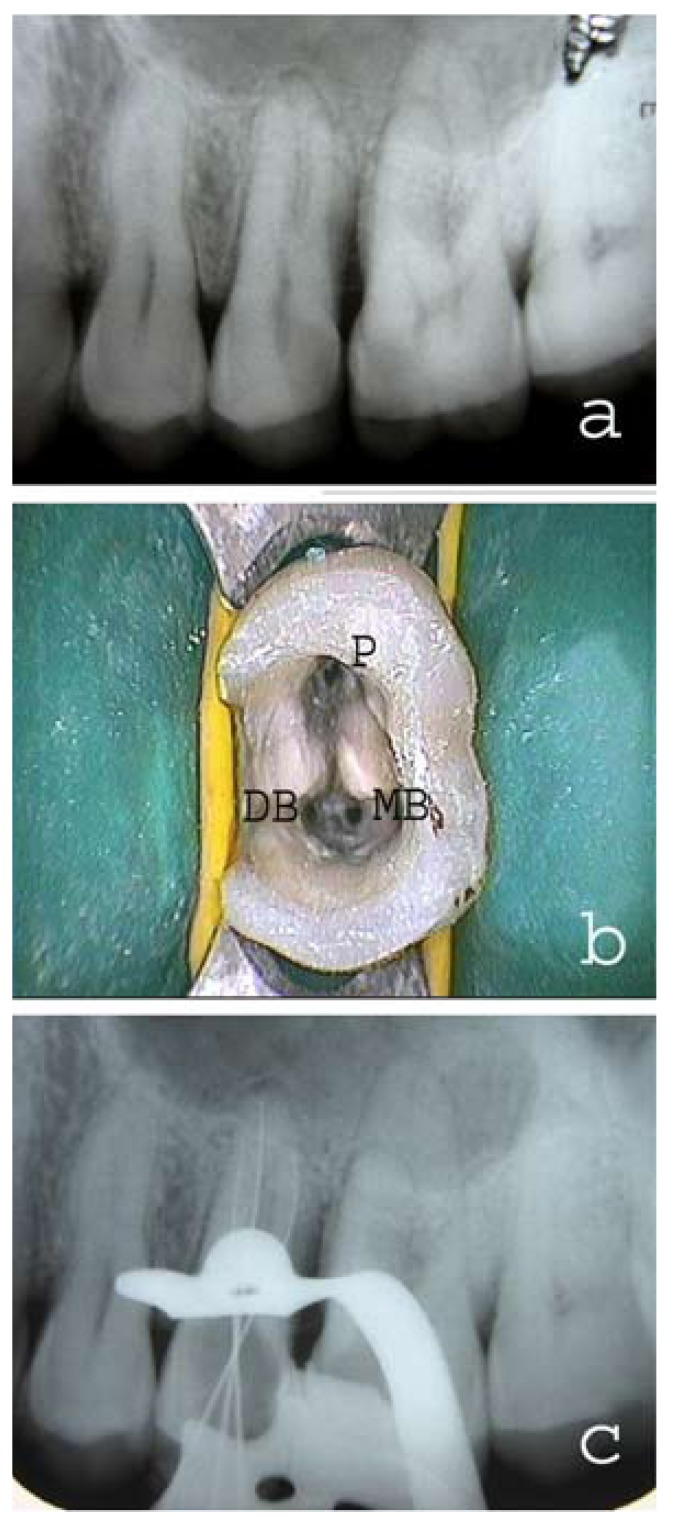
(a) Preoperative radiograph showing the presence of three separate roots in left maxillary second premolar. (b) The pulpal floor as seen after access opening. The three root canal orifices were located in the same way as in maxillary molar (P-Palatal, MB-Mesiobuccal, DB-Distobuccal). (c) Radiographic confirmation of working length.

**Figure 2:**
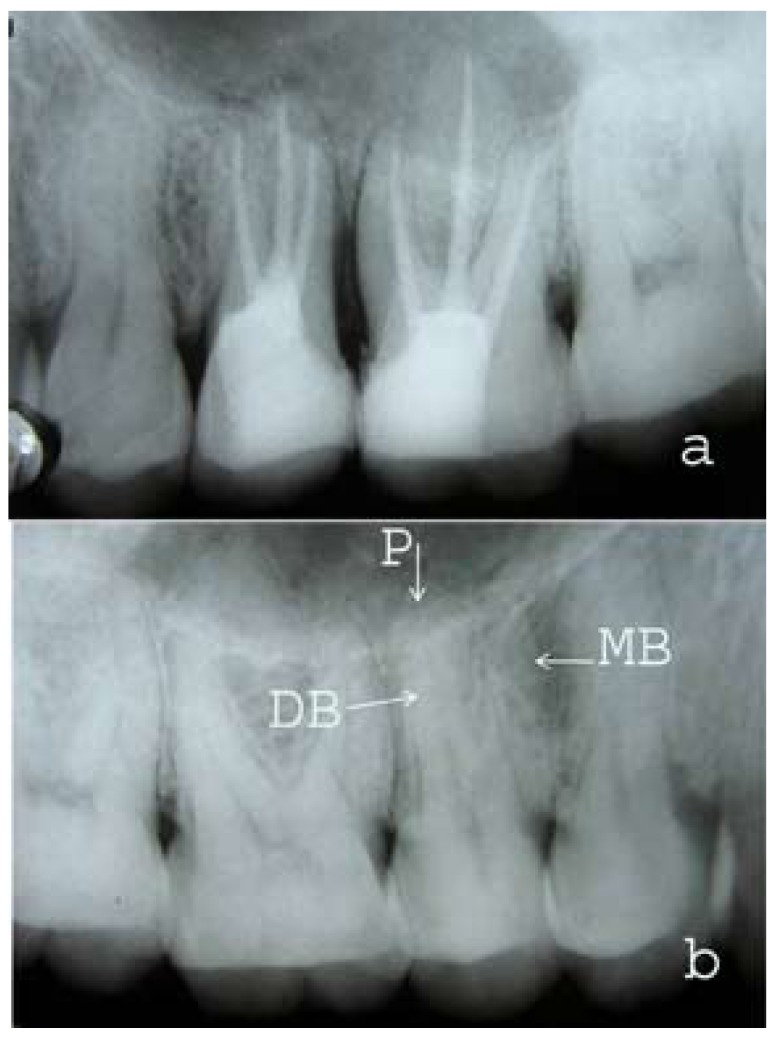
(a) Post-operative radiograph with all the three canals obturated in second premolar. (b) Radiograph showing the presence of three-rooted maxillary right second premolar in the same patient (arrows pointing; P-Palatal, MB-Mesiobuccal, DB-Distobuccal).

A 35 year old male patient with non-contributory medical history reported in the post-graduate clinics of our college and hospital, having pain in the left maxillary posterior region. The clinical examination showed large carious lesions in tooth #25 and 26 with pulp exposure. The patient was sensitive to percussion and palpation on both teeth. Vitality tests (hot, electric pulp test [EPT]) on the involved tooth showed abnormal responses indicating that irreversible pulpitis had occurred. Preoperative radiograph was taken which revealed deep carious lesions in tooth #25 and 26 approaching the pulp (Fig. 1). The radiograph also revealed a complex root canal anatomy in tooth #25. Three distinct roots were observed in second premolar resembling the maxillary first molar. The pulp chamber in tooth #25 was located more apically and also the mesiodistal width of crown was approximately equal to its mesiodistal width at the mid-root region. Nonsurgical endodontic treatment was planned in both #25 and 26 over two visits with the use of calcium hydroxide as inter-appointment, intra-canal medicament. After the administration of the local anesthetic, 2% Lignocaine with 1:100,000 epinephrine (Xicaine, ICPA Health Products Ltd, Gujarat, India), access cavity was prepared in tooth #25 under the rubber dam isolation. Access opening was given a ‘T’ shaped outline to locate all the three root canal orifices. On entry into the pulp chamber, three canal orifices were observed i.e., mesiobuccal, distobuccal and palatal, positioned as in maxillary first molars (Fig. 1). All the three root canal orifices were located using #10 K-file (Dentsply Maillefer, Ballaigues, Switzerland). Working length was established with radiograph by #15 K-files (Fig. 1) and confirmed using an Apex locator (Root ZX, J. Morita Inc). The canals were cleaned and shaped with hand K-files and nickel titanium rotary files (Protapers, Dentsply Maillefer, Switzerland) frequently irrigating with 5% sodium hypochlorite and 17% EDTA. Calcium hydroxide was used as an intracanal medicament and the access cavity was sealed with Cavit (ESPE, Seefeld, Germany). Cleaning and shaping was also performed in tooth 26 using K-files and nickel titanium rotary files (Protapers, Dentsply Maillefer, Switzerland) followed by placement of calcium hydroxide intracanal medicament. The access cavity in first molar was sealed with Cavit (ESPE, Seefeld, Germany). In the next appointment, one week later, the patient was asymptomatic. Intracanal calcium hydroxide dressing was removed from tooth #25 and the canals were irrigated with 5% sodium hypochlorite. Canals were dried with sterile paper points and obturated with gutta-percha and AH Plus sealer (Dentsply DeTrey GmbH, Germany) by cold lateral condensation technique. Access cavity was restored with composite resin. Tooth #26 was subsequently obturated with gutta-percha and AH Plus sealer (Dentsply DeTrey GmbH, Germany) using cold lateral condensation technique and restored with composite resin. Radiograph was taken to confirm the quality of the obturation (Fig. 2). Periapical radiograph was also taken in relation to right maxillary premolars in the same patient which revealed the presence three separate roots in tooth #15 (Fig. 2).

## Discussion

The variability of the root canal system of multi-rooted teeth represents a challenge to both endodontic diagnosis and treatment. Many of the challenges faced during root canal treatment may be directly attributed to an inadequate understanding of the tooth morphology. Undetected extra roots or root canals are a major reason for failure of root canal treatment. Endodontic success in teeth with a number and morphology of canals above that normally found requires a correct diagnosis and careful clinical radiographic inspection. Morphological variations in pulpal anatomy must be always considered before beginning treatment. Periapical radiographs should be studied carefully as they are good indicators for any morphologic or anatomic variations in a tooth. Accurate evaluation of pre-operative radiographs is essential to detect extra roots/canals (7). If some anatomic variations are suspected during the radiographic examination then it is preferred to take additional radiographs form mesial and distal angulations to get extra information. This type of maxillary premolars with three root canals, palatal, disto-buccal and mesio-buccal, very similar to the maxillary molar are often termed as ‘small molars’ or ‘radiculous’ (8). A general guideline for the identification of a three-rooted maxillary premolar on a straight-on preoperative radiograph is if the mesial-distal width of the mid-root image appears equal to or greater than the mesial-distal width of the crown image, then the tooth most likely has three canals. This guideline acts as a good visual clue, but is not absolute. Slight modification of the access cavity is required to locate the canal orifice in teeth with complex anatomy (9) A T-shaped outline to the access cavity is recommended to locate the root canal orifices in three-rooted maxillary premolars. Bilateral occurrence of complex root canal anatomy is rare, but we should always look for the anomalies in other teeth in the same patient. In this case also, the radiograph of right maxillary posterior region revealed the presence of three roots in right maxillary second premolar, hence the bilateral presence of the anomaly.

## Conclusion

Knowledge of the basic root canal anatomy and its variations from the normal is required for the success of non surgical root canal treatment. Careful interpretation of radiographs coupled with access refinement and inspection of the pulpal floor under magnification can play a very important role for endodontic management of complex root canal anatomy.
